# Case Report: Human Subcutaneous Sparganosis in a Thai Migrant

**DOI:** 10.4269/ajtmh.19-0456

**Published:** 2019-09-30

**Authors:** Veronika Muigg, Marie-Therese Ruf, Stefan Schwarzkopf, Simon Huang, Natalja Denisjuk, Anna Stürmann, Michael Ritzler, Rahel Wampfler, Sven Poppert, Andreas Neumayr

**Affiliations:** 1 Department of Medicine, Swiss Tropical and Public Health Institute, Basel, Switzerland;; 2 University of Basel, Basel, Switzerland;; 3 DermaZentrum Schaffhausen, Schaffhausen, Switzerland;; 4 IDP Institute for Dermatohistopathology, Zürich, Switzerland;; 5 Labormedizinisches Zentrum Dr. Risch, Buchs, Switzerland

## Abstract

Human sparganosis is a cestode infection which is neglected as a differential diagnosis outside endemic countries. Diagnosis and therapy may be challenging depending on the clinical presentation and anatomic localization. The disease manifests predominantly as subcutaneous nodule(s) or intracranial mass lesion(s). Infection is primarily acquired by ingesting raw or undercooked amphibian or reptile flesh or by drinking water containing copepods. We report an unusual case of subcutaneous *Spirometra erinaceieuropaei* sparganosis presenting with two nonmigratory nodules in close proximity to each other on the right thigh of a Thai woman living in Switzerland.

## CASE REPORT

**Figure 1.**
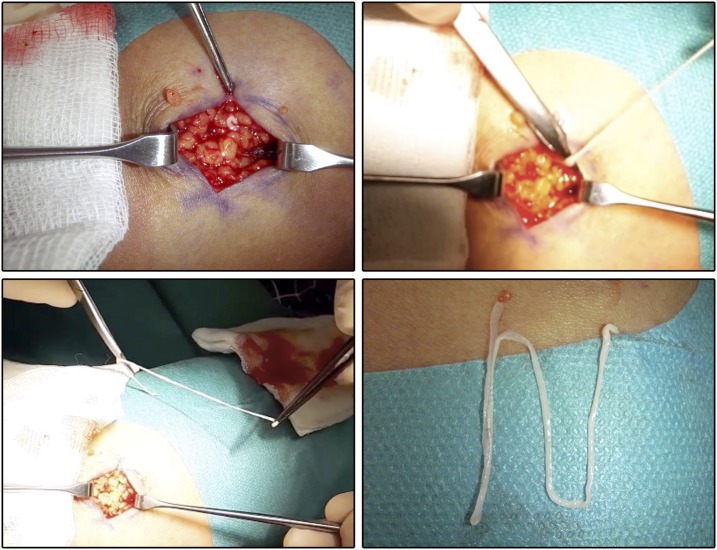
Intraoperative photographs showing the extraction procedure of the parasites. This figure appears in color at www.ajtmh.org.

In June 2018, a 55-year-old Thai woman living for the last 25 years in Switzerland presented with a history of two subcutaneous, painless, nonitching nodules to a dermatologist in Switzerland. The nodules had been present for 4 years, and the patient reported neither growth nor change of morphology over time. On palpation, two firm subcutaneous nodules of coarse consistency, both measuring approximately 4 cm in diameter, located on the right upper thigh in close proximity to each other, were identified. Neither signs of inflammation nor local lymphadenopathy was present, and the physical examination was otherwise unremarkable. Basic laboratory investigations (including differential blood count, C-reactive protein, and liver function tests) were all within normal limits. Because the clinical presentation suggested a benign soft tissue process, imaging was dispensed under the assumption that definite diagnosis would be established by surgical excision and histological examination. Hence, the patient was referred to a plastic surgeon. On incision of the first nodule, a whitish, thin, flattened, worm-like object (∼0.3 × 15 cm) was pulled out. On incision of the second nodule, a morphologically similar structure was removed (Figure 1). (Note: Because of the unexpected findings at incision of the first nodule, the second nodule was operated on 10 days later, after our laboratory had been consulted and a provisional diagnosis had been established.)

**Figure 2.**
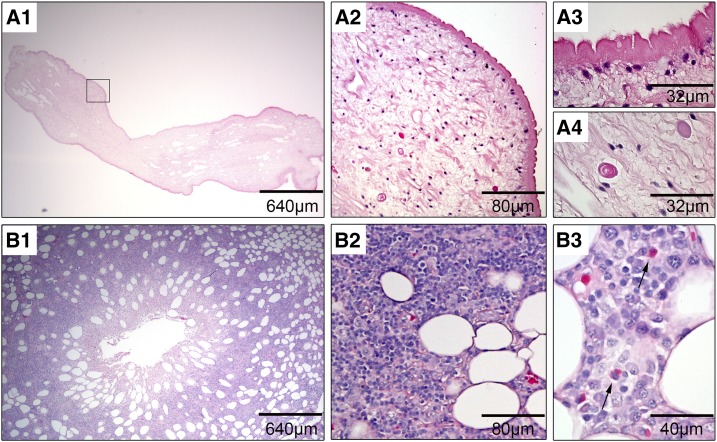
Histopathology of *Spirometra erinaceieuropaei* and the surrounding patient tissue. (**A**) Histopathology of *S. erinaceieuropaei*. Low-magnification image of one part of the helminth (**A1**). Higher magnification showing the presence of typical histopathological features of cestodes like the highly folded eosinophilic noncellular tegument (**A2** and **A3**) and the presence of calcareous corpuscles (**A2** and **A4**). (**B**) Surrounding human tissue showing the duct of the removed helminth in the center (**B1**). Chronic panniculitis (mixed infiltrate of lymphocytes, granulocytes, plasma cells, foamy macrophages, and giant cells) (**B2**), with few eosinophils (arrows, **B3**). Formaldehyde-fixed paraffin-embedded tissue sections were stained with hematoxylin–eosin. This figure appears in color at www.ajtmh.org.

**Figure 3.**
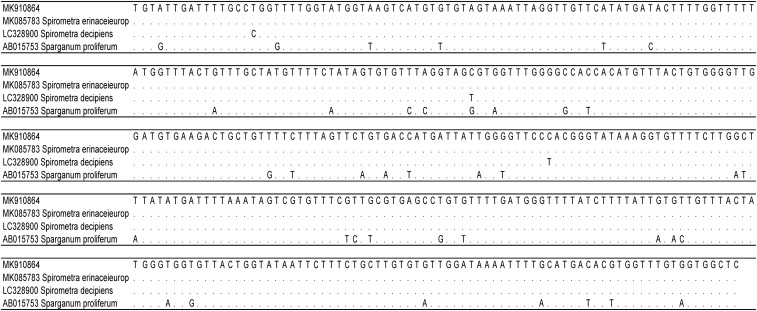
Alignment of the parasite’s mitochondrial cytochrome oxidase 1 nucleotide sequence with respective reference sequences. MK910864 = extracted parasite; MK085783 = *Spirometra erinaceieuropaei*; LC328900 = *Spirometra decipiens*; AB15753 = *Sparganum proliferum*.

Because the findings were suspicious of a helminth, two samples (one from the first resected worm fixed in formalin and the other one from the second resected worm stored in normal saline solution) were sent to the National Reference Center for Human Parasitic Diseases at the Swiss Tropical and Public Health Institute in Basel. Macroscopic and microscopic investigation of the sample stored in normal saline solution showed an amorphous structure exhibiting slow, undulating movements. An anterior end was identified by finding an oral opening of a helminth, whereas the posterior end was disrupted. A part of the sample was embedded in paraffin and used for histological investigation, which revealed typical characteristics of spargana: a folded cuticula with microvilli and calcareous corpuscles (Figure 2). For species determination, DNA was isolated from another part and a sequence of the mitochondrial cytochrome oxidase (COX1) gene was amplified and sequenced as described elsewhere.^1^ A 398–base pair-long sequence of the COX1 was determined (GenBank accession number MK910864) showing 100%, 99.25%, and 90.20% sequence homology to *Spirometra erinaceieuropaei* (no single nucleotide polymorphisms [SNPs]), *Spirometra decipiens* (3 SNPs), and *Sparganum proliferum* (> 35 SNPs), respectively (Figure 3)*.* Thus, the helminth was identified as *S. erinaceieuropaei*. The healing of the wounds proceeded without complications. No oral anthelminthic treatment was administered, and no subsequent lesions occurred since.

## DISCUSSION

Sparganosis is a rare zoonotic cestode infection caused by the second-stage larva of various species of the pseudophyllidean tapeworm *Spirometra* spp. In the Old World, *S. erinaceieuropaei* causes most infections, whereas *Spirometra mansonoides* is mostly prevalent in the New World.^2,3^

Approximately 1,600 human cases have been reported worldwide since the first case description in 1882 in Xiamen, Fujian Province, China. More than 80% of all cases have been reported from China, followed by Korea, Thailand, and the United States.^4^

**Figure 4.**
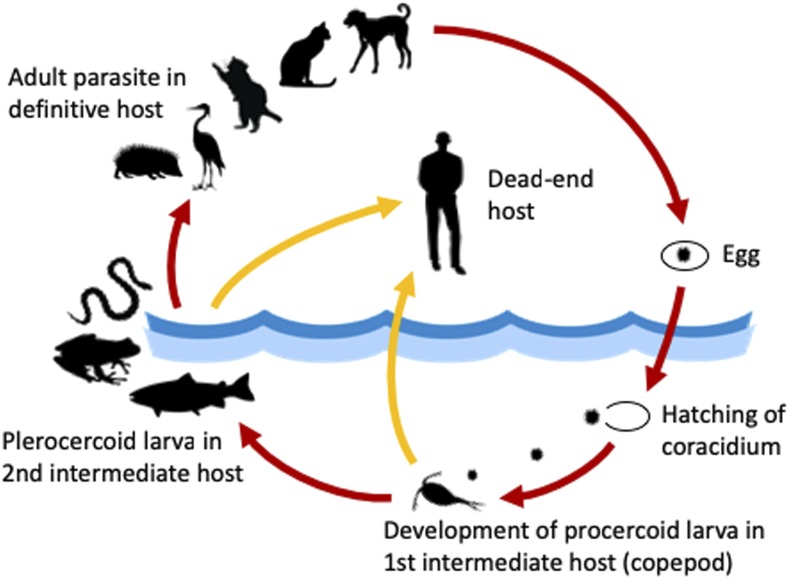
Life cycle of *Spirometra* spp. This figure appears in color at www.ajtmh.org.

The parasite’s life cycle is similar to that of the fish tapeworm *Diphyllobothrium* spp. Carnivorous mammals serve as definite hosts, harboring the adult worm in the intestine and shedding eggs in their feces. When eggs get into freshwater, they release coracidia. Coracidia are ingested by copepods, the first intermediate host, and develop into procercoid larvae (first-stage larvae) within 3–11 days.^5^ When the copepod is ingested by fish, reptiles, or amphibians, the second intermediate hosts, procercoid larvae develop into plerocercoid larvae (second-stage larvae, “sparganum”), lodging in various body tissues. When the second intermediate host is ingested by the definite host, plerocercoid larvae develop into adult worms in its intestine. In addition, several predator species may serve as paratenic hosts (Figure 4). Humans are accidental hosts and become infected by ingesting raw or undercooked second intermediate hosts or by drinking untreated water contaminated with infected copepods. In some Asian countries, the application of poultices, made from second intermediate hosts, on skin or eyes has been reported as an alternative source of infection.^4^

Usually, humans are dead-end hosts in which larvae do not reach patency. However, in rare cases, eggs have been found in human feces.^6^

The incubation period in humans varies from several days up to years.^7^ The clinical picture depends on the final destination of the migrating spargana. Subcutaneous tissue, as well as abdominal viscera, the eye, and the central nervous system (CNS), may be infiltrated by the larvae and manifest as nodular mass lesions. A review from Korea revealed the subcutaneous tissue and the CNS as the most frequently infiltrated anatomic sites, accounting for 49.9% and 36.2% of cases, respectively.^8^

The patient described in our case frequently travels to her home country Thailand, where at least 62 cases have been reported in the past 60 years. In most cases, sparganosis presents as a single subcutaneous lesion. Of note, ocular involvement due to applying frog flesh poultices in traditional medicine was described in one-third of cases in Thailand up to the 1990s. Since then, the number of such cases has decreased, probably because of increasing disappearance of this practice.^9,10^ In general, reports on multiple lesions, like in our case, are rare. Case reports like that of a Korean woman with a history of eating snakes 40 years before developing multiple masses on different body sites (e.g., breast, elbow, and thigh) are exceptional.^11^

A differential diagnosis to be kept in mind for prognostic reasons would be infection with *Spirometra proliferans*, where multiple masses may occur because of the worm’s ability of branching and budding, resulting in a more serious clinical outcome.^3,4^

Multiple infections may also occur in case of local inoculation of the larvae due to applying poultices made out of reptile or amphibian flesh. However, our patient denied having used this form of traditional medicine.

The patient also denied ingesting raw or undercooked reptiles or amphibia. However, she reported drinking filtered tap water at her hometown in Thailand. Thus, the most likely route of infection remains drinking copepod-contaminated water, as filtration might have been insufficient. A review highlighted ingestion of snakes or frogs observed in 63.4% of the presented cases, whereas drinking unfiltered water accounted for 16.9% as the suspected infection route.^8^ Wiwanitkit identified the history of drinking “impure” water as the main risk factor for human sparganosis in Thailand.^12^

Blood eosinophilia, usually suggestive of a tissue-invasive helminth infection, was absent in our patient. This is in line with observations by Zhang et al.,^13^ who found eosinophilia in only 30% of sparganosis cases.

The initial differential diagnoses in our case were limited to sebaceous cysts, fibroma, and lipoma because typical characteristics of tissue-invasive helminth infections, such as creeping eruption/migratory swelling or blood eosinophilia, were absent. When such characteristics are present, differential diagnoses primarily depend on epidemiology and exposure. In the case of nonmigrating lesions, dirofilariasis or soft tissue cysticercosis could also be considered especially because blood eosinophilia is absent in most of these cases too.^14^

Identification of *S. erinaceieuropaei* in our case is in line with two case series from Thailand where genotyping revealed *S. erinaceieuropaei* in all 12 investigated cases.^9,10^ The diagnosis is usually made by histological identification followed by molecular confirmation of the parasite, after surgical excision. When surgery is not feasible, serology and imaging remain important diagnostic tools. Although the use of highly sensitive serological assays has been described in publications from endemic regions, serological testing remains largely unavailable in most nonendemic settings.^15^ In the case of subcutaneous sparganosis, a band-like structure may be seen on magnetic resonance imaging. Ultrasonography may show a coiled linear hypoechoic body in a clear oval mass.^16^

Species identification is desirable so as not to miss *S. proliferum* infections, which may show continued growth in the case of incomplete removal. Histological identification of *Sparganum* spp. is based on the finding of a characteristic deep-folded tegument and calcareous corpuscles which are unique to cestode tissue.^17^ However, in this developmental stage of the parasite, a definitive species diagnosis can only be achieved by molecular methods. Up to now, no standards for molecular identification are defined. Most commonly, the mitochondrial *COX1* gene is amplified and sequenced. In this context, it needs to be noted that amplification and sequencing is based on intact DNA and that formalin (which causes severe DNA damage) should, therefore, be avoided for sample preservation in such cases.

Treatment of sparganosis is primarily based on surgical removal of the larva(e). No medical treatment standard has been defined for patients suffering from masses not suitable for surgical resection. However, high-dose praziquantel (repeated cycles of 50–75 mg/kg body weight/day for 10 days) has been reported to be effective in inoperable cases of cerebral sparganosis.^13,18^

In summary, human infections with *Spirometra* spp. are rare, usually not considered outside endemic regions, and as in the case presented, diagnosis is only made after surgical removal of the parasite. There is a lack of commercially available serological tests, and species identification requires sequencing.

## Supplemental video

Supplemental materials
